# Individual, household and national factors associated with iron, vitamin A and zinc deficiencies among children aged 6–59 months in Nepal

**DOI:** 10.1111/mcn.13305

**Published:** 2021-12-13

**Authors:** Stanley Chitekwe, Kedar Raj Parajuli, Naveen Paudyal, Karan Courtney Haag, Andre Renzaho, Abukari Issaka, Kingsley Agho

**Affiliations:** ^1^ Nutrition Section United Nations Children's Fund (UNICEF) Kathmandu Nepal; ^2^ Nepal Ministry of Health and Population Kathmandu Nepal; ^3^ School of Social Sciences and Psychology Western Sydney University Penrith New South Wales Australia; ^4^ School of Science Western Sydney University Penrith New South Wales Australia; ^5^ School of Health Sciences Western Sydney University Penrith New South Wales Australia

**Keywords:** ferritin, hidden hunger, iron deficiency, Nepal National Micronutrient Status Survey, vitamin A, zinc

## Abstract

Iron, vitamin A and zinc deficiencies are the top three micronutrients contributing to disability‐adjusted life years globally. The study assessed the factors associated with iron, vitamin A, and Zinc deficiencies among Nepalese children (*n* = 1709) aged 6–59 months using data from the 2016 Nepal National Micronutrient Status Survey. The following cut‐off points were applied: iron deficiency [ferritin < 12 μg/L or soluble transferrin receptor (sTfR) > 8.3 mg/L], vitamin A deficiency (retinol‐binding protein < 0.69 μmol/L) and zinc deficiency (serum zinc < 65 μg/dl for morning sample and <57 μg/dl for afternoon sample). We used multiple logistic regression adjusted for sampling weights and clustering to examine the predictors of micronutrient deficiencies. The prevalence of iron depletion (ferritin), tissue iron (sTfR), vitamin A and zinc deficiencies were 36.7%, 27.6%, 8.5% and 20.4%, respectively. Children were more likely to be iron deficient (ferritin) if aged 6–23 months, stunted, and in a middle‐wealth quintile household. Vitamin A deficiency was associated with development region and was higher among children living in severe food‐insecure households and those who did not consume fruits. Zinc deficiency was higher among children in rural areas and the poorest wealth quintile. The Government of Nepal should focus on addressing micronutrient deficiencies in the early years, with emphasis on improving food systems, promote healthy diets, among younger and stunted children and provide social cash transfer targeting high‐risk development regions, poorest and food insecure households.

## Key messages

• This study examined risk factors and concurrent micronutrient deficiencies in children aged 6–59 months in Nepal.
• The likelihood of vitamin A deficiency was significantly higher in children from households who reported severe food insecurity compared with those in food‐secure households.
• The odds of being zinc deficiency were significantly higher among rural children compared with their urban counterparts.
• Existing and potential interventions to alleviate iron deficiency among Nepalese children should target geographical and ethnic differentials.
• Encouraging the government to improve food systems, promote healthy diets among younger and stunted children, and provide social cash transfer targeting high‐risk development regions and poorest and food‐insecure households.

## Graphical abstract

Individual, household and national risk factor for hidden hunger among under 5 s in Nepal.

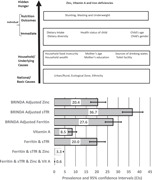

## 1 INTRODUCTION

Hidden hunger (otherwise known as micronutrient deficiencies) is a significant public health concern globally. It affects two billion people, predominantly young children and women of reproductive age (Gödecke et al., 2018; Hodge, 2016). Iron, vitamin A and zinc deficiencies are the most widespread forms of hidden hunger and are the top three micronutrient deficiencies in the world in terms of disability‐adjusted life years (DALY); they account for 65.8%, 15.8%, and 12.5% of all DALYs lost due to hidden hunger (Gödecke et al., 2018).

Iron deficiency is the most common cause of anaemia (62.6%) and anaemia‐related disability globally, accounting for 36.6 million or 59.5% of years lived with disability (YLDs) due to anaemia (Kassebaum, 2016). Iron deficiency increases morbidity and mortality and impairs mental and motor development in children (Lynch et al., 2018). Vitamin A deficiency affects 29% of children aged 6–59 months (Stevens et al., 2015) and accounts for 10% of YLDs in children under 5 years (Global Burden of Disease Study, 2020). It is a leading cause of preventable childhood blindness, impairs immune function and increases the risk of child mortality from diarrhoea, measles and other infectious diseases (Holick, 2007; Stevens et al., 2015). Zinc deficiency affects an estimated 17% of the world's population and 30% of South Asians (Wessells et al., 2012). In young children, zinc deficiency impairs linear growth, immunity and resistance to infectious diseases such as diarrhoea and pneumonia (Brown et al., 2004).

Despite the impact of hidden hunger on child survival and development, few studies have focused on identifying risk factors that can be modified. There have been some studies on VA (Gorstein et al., 2003) and iron (Siegel et al., 2006), and zinc deficiencies (Basnet et al., 2012) among Nepalese Children. These studies have had one of the following limitations: either dated (e.g., based on the 1998 Nepal National Micronutrient survey) or geographically specific, limiting their generalizability at the national level.

We conducted this study to investigate preventable factors associated with deficiencies of iron, vitamin A and Zinc in children aged 6–59 months in Nepal using data from the 2016 Nepal National Micronutrient Status Survey (NNMSS). Our study identifies risk factors using the hidden hunger analytical framework (Gödecke et al., 2018), adopted from the UNICEF conceptual framework on causes of malnutrition. Findings from this study will inform policy and programme responses to control micronutrient deficiencies. Unexpected study outcomes will help propose further research into unique circumstances that explain the findings, thus further defining the boundaries of knowledge of preventable factors associated with micronutrient deficiencies.

## 2 METHODS

### 2.1 Data source

The NNMSS 2018 was the source of data for this study. New ERA conducted the NNMSS during the dry season from 1 April to 25 June 2016, in collaboration with the Ministry of Health and Population (MoHP) of Nepal, the United States Agency for International Development (USAID), the United Nations Children's Fund (UNICEF) Nepal and the United States Center for Disease Control and Prevention (CDC) Atlanta. The NNMSS was a cross‐sectional survey that provided estimates of micronutrient deficiencies at the national level and for Nepal's geographic regions (Eastern, Central, West, Midwest and Far West) and ecological zones [Terai (plains), Hills, and Mountains]. The present analysis used a sample of 1709 children aged 6–59 months.

We collected data using stratified multistage cluster sampling without replacement. We selected 180 clusters from 15 strata with probability proportional to size. A total of 24 households were selected from each cluster by systematic sampling (*n* = 4320). After enumerating all children 6–59 months (henceforth referred to as children), 12 children were randomly selected from households selected from each cluster. The survey sought to collect data from 2160 children; based on sample size calculated assuming 46% anaemia prevalence based on the 2011 DHS, aiming for a 3.5% precision, a design effect of 2.25, and household and individual response rates of 95% and 90%, respectively (Ministry of Health and Population—MOHP/Nepal New ERA/Nepal & ICF International, 2012). Full details about the study area, study population, and sampling strategy are available in the NMSS Report (Ministry of Health Nepal et al., 2018). Of the 2160 children planned for data collection, 1728 children were available in the selected clusters. Of those, 1709 (98.9%) completed the interview (*n* = 5 refusals and *n* = 14 were unreachable). The present analysis used a sample of 1709 children aged 6–59 months.

### 2.2 Data collection

#### 2.2.1 Procedure

An 11 ml sample of venous blood was collected from children by trained phlebotomists.

All the equipment and supplies needed for specimen processing and storing were set up in appropriate locations in all 180 cluster. Bio‐specimens (blood, stool and urine) were collected in the field from all clusters. Maintaining the temperature between 2°C and 8°C, fresh blood samples were transported to the National Public Health Laboratory (NPHL) within seven days of sample collection. An anticoagulant was not needed in tubes where serum was collected. The Lab Technicians centrifuged the blood sample and separated Serum and Plasma. The processed specimens were stored in cryovial boxes and placed into a portable freezer until they were transferred to the district cold chain to be stored at −20°C. Serum, plasma and urine were stored in −86°C freezers in NPHL, half of which were shipped to the international laboratories in China, Germany and Guatemala, while the remaining half were stored as back‐up samples (Ministry of Health Nepal et al., 2018). VitMin Lab analysed serum C‐reactive protein (CRP), ɑ−1‐acid glycoprotein (AGP), ferritin, transferrin receptor (sTfR), and retinol‐binding protein (RBP) with a sandwich ELISA (Erhardt et al., 2004). Because no values were available for sTfR, a commercially available kit (Ramco Laboratories) was used to measure the concentration of sTfR (8.93 mg/L). To get calibration curves in the physiological range a dilution scheme was used. The VitMin Lab uses an in‐house Sandwich ELISA thus no kit name or batch number can be listed (Erhardt et al., 2004).

Zinc was analysed using atomic absorption flame emission spectroscopy (Ministry of Health Nepal et al., 2018). Ferritin, sTfR, RBP and serum zinc are influenced by the inflammatory process, leading to either over or underestimated deficiency. The NNMS, therefore, collected data on biomarkers of inflammation [αAcid glycoprotein (AGP) and C‐Reactive Protein (CRP)]. Adjustments for the influence of inflammation on these biomarkers were made using the approach recommended by the Biomarkers Reflecting Inflammation and Nutritional Determinants of Anemia (BRINDA) working group (Namaste et al., 2017). We adjusted for inflammation of ferritin (sTfR), (RBP) and serum Zinc by regression to a pooled country reference with CRP and AGP (ferritin, RBP,  Zinc) or AGP only (sTfR) (Namaste et al., 2017).

#### 2.2.2 Outcomes

The outcome variables for micronutrient deficiencies in children aged 6‐59 months were iron deficiency using two indicators; (a) (adjusted ferritin <12.0 μg/L)—measure of the amount of iron in body stores if there is no concurrent infection: higher concentrations show the size of the iron store; when the concentration is low then iron stores are depleted (Worwood, 2007), and (b) adjusted sTfR >8.3 mg/L)—is derived mainly from developing RBCs and so reflects the magnitude of erythropoiesis and the increased demand for iron; the concentration rises in iron deficiency anaemia and it is an indicator of the severity of iron insufficiency only when iron stores have been exhausted, provided that there are no other causes of abnormal erythropoiesis (Beard, 2007), vitamin A deficiency (adjusted RBP < 0.69 μmol/L) and zinc deficiency (adjusted serum zinc < 65.0 μg/dl for morning non‐fasting blood samples and <57.0 μg/dl for afternoon non‐fasting blood sample) (McDonald et al., 2020; Namaste, Aaro, et al., 2017; Namaste, Rohner, et al., 2017; Rohner et al., 2017). The regression‐adjustment equations for the inflammation carried out by the VitMin Lab are indicated in the hyperlink.

#### 2.2.3 Covariates

The NNMSS used structured questionnaires to obtain information from mothers or caregivers of children aged 6–59 months. The information collected included age, sex, consumption of seven food groups 24 h before the survey. A 2‐week recall of fever, cough, and diarrhoea was collected. Anthropometric measurements, namely weight and height, were taken. We documented the biological samples collected. Enumerators collected information on the child's mother (including age, education); and household assets, water and sanitary facilities, and residence (Ministry of Health Nepal et al., 2018).

**Figure 1:**
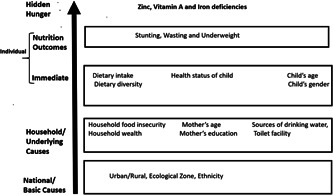
Determinants of hidden hunger used in the study based on the modified conceptual framework on causes of malnutrition (UNICEF, 1990) using variable available in the NNMSS. The basic factors are considered national level while the nutrition outcomes and immediate factors are considered household level and underlying factors are considered household level

We selected covariates for this study based on the hidden hunger conceptual framework adapted from the UNICEF conceptual framework for improving the nutrition of children and women in developing countries (UNICEF, 1990) and previous studies on micronutrients deficiencies (Cappellini et al., 2020; Gibson, 2006; Rahman et al., 2017). We categorized selected covariate variables into four groups: (i) basic factors (development region, ecological region, urban/rural, and ethnicity), (ii) underlying factors/household factors (household food security, household wealth, type of toilet facility, and source of drinking water), (iii) immediate factors/individual factors [health status factors, dietary intake, dietary diversity (DD), child's age and gender] and (iv) nutritional factors (anthropometric and nutritional status) as shown in Figure 1. We considered basic factors as national level, while underlying factors are considered household level, and both immediate and nutrition outcomes are considered individual level.

We calculated the household wealth index using the principal components analysis method. We categorized the wealth index into five quintiles: lowest, second, middle, fourth and highest. The five quintiles were divided into five equal parts, each being 20% and the bottom 20% of was referred to as the lowest quintile, and the top 20% as the highest quintile (Ministry of Health Nepal et al., 2018). We used the poorest as the reference population based on previous research (Moschovis et al., 2018) and for easy epidemiological interpretations.

Household food insecurity (HFI) was calculated by summing up the seven HFI (access) frequency questions with scores ranging from 0 to 27. Households were categorized into four groups, namely: food secure (0), mildly food insecure (1–2), moderately food insecure (3–10), and severely food insecure (more than 10) (Coates et al., 2007). Toilet facilities were categorized into flush or pour‐flush toilets and pit latrines. The unimproved water source was any source other than piped water, tube well borehole, protected well or spring, Public tap/standpipes, rainwater or bottled water (World Health Organization, 2017). DD was calculated by summing the 8 food groups consumed during the last 24 h. These food groups are grains roots and tubers, legumes and nuts, milk/dairy products, fresh foods (meat, fish, poultry and liver/organ meats), vitamin A‐rich fruits and vegetables, other fruits and vegetables, eggs and breast milk, and was categorized into the child had ≥5 food groups, and the child had <5 food groups (World Health Organization, 2010).

### 2.3 Statistical analysis

STATA/MP version 14 (Stata Corp.) was used for statistical analysis. We used the “Svy” commands to adjust for the cluster‐sampling design and weight. We conducted frequency tabulations to describe the characteristics of children and their households and used the Taylor series linearization method to estimate the 95% confidence intervals (CIs) of the prevalence of micronutrient deficiencies.

We then conducted bivariate and multivariate logistic regression to identify significant determinants of micronutrient deficiencies (*p* < 0.05), adjusted for clustering and sampling weights. The multivariate analysis involved four‐stage modelling. The basic factors were entered into the first stage model. We conducted a manually executed elimination method to determine factors associated with vitamin A, iron and zinc deficiencies at a 0.05 significance level. The significant factors in the first stage were added to the underlying factors in the second stage model; the elimination procedure then followed this. We used a similar approach for immediate and nutrition factors in the third and fourth stages, respectively. In the final model, we tested and reported any co‐linearity. We reported unadjusted and adjusted odds ratios (OR) with 95% CIs.

### 2.4 Ethical consideration

The Nepal Health Research Council (NHRC) granted ethical clearance for the NNMSS. Mothers or caregivers provided consent on behalf of their participating children. Enumerators explained to mothers or caregivers the survey goals, procedures, risks and benefits, and how participation in the survey contributes to public health. Written informed consent was obtained from literate mothers or caregivers, whereas oral informed consent, coupled with the witnesses' signatures, was obtained from illiterate interviewees. We obtained permission from the Ministry of Health and Population of Nepal to use the NNMSS data set for this study.

## 3 RESULTS

### 3.1 Selected characteristics of the children and their households

Of the 1709 children aged 6–59 months included in the study, 54% were male, residence in rural areas (87%), and over 35% belonged to moderately or severely food insecure households (Table 1). Malnutrition was high with 36% stunting and 12% wasting, and 20% of children had diarrhoea and over one‐third had a fever or cough two weeks before the survey. Only 49% of children consumed a minimum diet diversity (at least four out of seven food groups). The consumption of provitamin A‐rich fruits was 61%, while that of eggs was only 12% in the last 24 h.

**Table 1:** Characteristics of Nepalese children aged 6–59 months and their households (*N* = 1709)

Characteristics	(%)
*Basic factors*	
Ethnicity (caste)	
Brahmin/Chettri	30.3
Dalit	18.4
Janajati	29.2
Others^b^	22.1
Residence	
Urban	13.1
Rural	86.9
Geographic region	
Eastern	21.6
Central	36.5
Western	17.6
Mid‐western	14.2
Far‐western	10.1
Ecological zone	
Mountain	7.7
Hill	41.4
Terai	50.9
*Underlying determinants*	
Household Wealth Index	
Lowest	21.5
Second	19.4
Middle	19.3
Fourth	20.5
Highest	19.3
Household food insecurity	
Food secure	53.4
Mild food insecurity	10.8
Moderate food insecurity	27.4
Severe food insecurity	8.4
Sources of drinking water	
Improved	93.7
Unimproved	6.3
Type of toilet facility	
Flush or pour‐flush toilet	71.3
Pit latrine	28.7
Relation to child	
Biological parents	96.5
Others^a^	3.5
Mother's education	
No schooling	23.2
Primary	18.6
Secondary education or more	58.2
Mothers/caretakers age (in category)	
16–30 years	46.9
31–40 years	20.4
40+ years	32.7
*Immediate determinants*	
Child's age	
6–23 months	30.5
24–59 months	69.6
Child's sex	
Male	53.7
Female	46.3
*Health status of child in the last 2 weeks*	
Fever	36.6
Cough	38.3
Diarrhoea	19.6
*Dietary intake in the previous day*	
Grains, roots and tubers	98.4
Legumes and nuts	73.5
Dairy products	48.8
Flesh foods	26.7
Eggs	11.7
Vitamin A‐rich fruits and vegetables	61.2
Other fruits and vegetables	30.8
Breast milk	28.1
Dietary diversity (0–8)	
≥5 food groups	28.2
<5 food groups	71.8
*Nutrition factors*	
Child stunted (height‐for‐age *z*‐score < −2 SD)	35.6
Child underweight (weight‐for‐age *z*‐score < −2 SD)	29.0
Child wasted (weight‐for‐height *z*‐score < −2 SD)	11.7
Body mass index (BMI)‐for‐age *z*‐score < −2 SD)	9.3

^a^ Grandmother, father, others.

^b^ Other Terai Caste, Newar, Muslim and others.

### 3.2 The prevalence of micronutrient deficiencies

**Figure 2:**
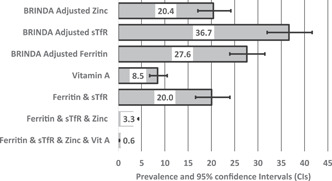
Prevalence and 95% confidence interval concentrations of micronutrients, one, two and three micronutrients status among children aged 6–59 months in Nepal

The prevalence of iron deficiency was 27.6% based on ferritin concentration and 36.7% based on sTfR concentration (Figure 2). The prevalence of vitamin A deficiency was 8.5% based on RBP concentration, and the prevalence of zinc deficiency was 20.4% based on serum zinc concentration. The prevalence of having one micronutrient (ferritin and sTfR) deficiency was 20%, and the prevalence of two micronutrients (ferritin, sTfR and Zinc) and all three deficiencies (ferritin; sTfR, Zinc and vitamin A) was 3.3% and 0.6%, respectively (see Figure 2). We provided in Table S1 the mean, standard deviation and lower quartile (<25th percentile), middle quartile (≥25th percentile and ≤75th percentile) of ferritin and sTfR, vitamin A.

We conducted a sub‐analysis on the relationship between DD (≥5 food groups out of 8 food groups) by the micronutrient status and socioeconomic factors. Results indicated that children from high socioeconomic status were more likely to consume five or more food groups. There was a significantly increased odds of iron deficiency among children who consumed five or more food groups using ferritin and sTfR concentration as the biomarker of iron status. There was a decreased odds of vitamin A deficiency among children who consumed five or more food groups (see Table S2 for details).

### 3.3 Factors associated with iron, vitamin A and zinc deficiency

Factors associated with iron deficiency, vitamin A deficiency and zinc deficiency in children aged 6–59 months are presented in Tables 2, 3, 4, respectively.

**Table 2:** Factors associated with iron deficiency among children aged 6–59 months in Nepal

Characteristic	Unadjusted OR (95% CI)	*p*‐value	Adjusted OR (95% CI)	*p*‐value
*Iron deficiency (ferritin concentrations)*				
Ethnicity (caste)				
Janajati	1.00		1.00	
Dalit	1.99 (1.36, 2.94)	0.001	1.59 (0.99, 2.54)	0.052
Brahmin/Chettri	2.01 (1.35, 2.99)	0.001	1.84 (1.18, 2.86)	0.008
Others^$^	2.99 (1.97, 4.53)	<0.001	2.08 (1.40, 3.08)	<0.001
Child's age (months)				
24–59	1.00		1.00	
6–23	3.62 (2.73, 4.80)	<0.001	3.99 (2.91, 5.48)	<0.001
*C*hild stunted (<−2 SD)				
No	1.00		1.00	
Yes	1.32 (1.00, 1.74)	0.047	1.61 (1.18, 2.18)	0.003
Household Wealth Index				
Lowest	1.00		1.00	
Second	0.98 (0.55, 1.77)	0.965	0.92 (0.52, 1.62)	0.766
Middle	1.89 (1.18, 3.05)	0.009	1.64 (1.03, 2.61)	0.039
Fourth	1.05 (0.67, 1.66)	0.814	0.98 (0.61, 1.58)	0.926
Highest	1.48 (0.78, 2.83)	0.227	1.45 (0.80, 2.64)	0.221
*Iron deficiency (sTfR concentrations)*				
Ecological region				
Mountain	1.00		1.00	
Hill	1.41 (0.87, 2.29)	0.156	1.42 (0.87, 2.33)	0.162
Terai	3.41 (2.05, 5.67)	<0.001	3.48 (2.02, 5.99)	<0.001
Child's age (months)				
24–59	1.00		1.00	
6–23	4.11 (3.10, 5.44)	<0.001	4.80 (3.57, 6.46)	<0.001
Child stunted (<−2 SD)				
No	1.00		1.00	
Yes	1.26 (0.96, 1.66)	0.089	1.69 (1.20, 2.38)	0.003
Household Wealth Index				
Lowest	1.00		1.00	
Second	1.34 (0.95, 1.89)	0.098	1.19 (0.79, 1.79)	0.397
Middle	2.77 (1.79, 4.27)	<0.001	1.92 (1.16, 3.17)	0.012
Fourth	1.46 (0.98, 2.16)	0.06	1.03 (0.64, 1.65)	0.912
Highest	1.63 (0.89, 2.96)	0.11	1.25 (0.72, 2.17)	0.419

Abbreviations: CI, confidence interval; OR, odds ratio; SD, standard deviation; sTfR, soluble transferrin receptor.

**Table 3:** Factors associated with vitamin A deficiency among children aged 6–59 months in Nepal

Characteristic	Unadjusted OR (95% CI)	*p*‐value	Adjusted OR (95% CI)	*p*‐value
*Development region*				
Western	1.00		1.00	
Far‐Western	2.22 (0.84, 5.64)	0.093	1.91 (0.79, 4.68)	0.175
Central	3.67 (1.44, 9.37)	0.007	3.41 (1.38, 8.41)	0.009
Mid‐Western	3.47 (1.41, 8.55)	0.007	2.82 (1.14, 6.98)	0.025
Eastern	3.59 (1.49, 8.71)	0.005	3.52 (1.49, 8.35)	0.034
*Consumed other fruits*				
Yes	1.00		1.00	
No	2.15 (1.23, 3.78)	0.008	2.91 (1.05, 3.48)	0.034
*Household food insecurity*				
Food secure	1.00		1.00	
Mild food insecurity	0.78 (0.38,1.58)	0.476	0.72 (0.35,1.48)	0.372
Moderate food insecurity	1.71 (0.96, 3.07)	0.070	1.62 (0.90, 2.93)	0.107
Severe food insecurity	2.35 (1.32, 3.83)	0.003	2.02 (1.15, 3.54)	0.015

Abbreviations: CI, confidence interval; OR, odds ratio.

**Table 4:** Factors associated with zinc deficiency among children aged 6–59 months in Nepal

Characteristic	Unadjusted OR (95% CI)	*p*‐value	Adjusted OR (95% CI)	*p*‐value
*Residence*				
Urban	1.00		1.00	
Rural	3.02 (1.56, 5.92)	0.001	2.69 (1.26, 5.77)	0.011
*Household wealth index*				
Lowest	1.00		1.00	
Second	0.44 (0.31, 0.63)	<0.001	0.45 (0.31, 0.64)	<0.001
Middle	0.58 (0.38, 0.88)	0.011	0.60 (0.40, 0.93)	0.022
Fourth	0.43 (0.27, 0.69)	0.001	0.49 (0.30, 0.76)	0.018
Highest	0.45 (0.25, 0.79)	0.002	0.59 (0.31, 1.25)	0.106

Abbreviations: CI, confidence interval; OR, odds ratio.

There was an increased odds of iron deficiency (ferritin concentration) among children belonging to the Brahmin/Chettri ethnic groups (adjusted odds ratio [AOR] = 1.84; 95% CI [1.18, 2.86]) and other ethnic groups (Newar, other Terai caste and Muslims) (AOR = 2.08; 95% CI [1.40, 3.08]) compared with Janajati ethnic group. In addition, the risk of iron deficiency was significantly higher among children aged 6–23 months than 24–59 months (AOR = 3.99; 95% CI [2.91, 5.48]); stunted children (AOR = 1.61; 95% CI [1.18, 2.18]); and children in middle wealth quintile households compared with the poorest households (AOR = 1.64; 95% CI [1.03, 2.61]). Using sTfR concentration as a biomarker of iron status, an increased odds of iron deficiency was found among children aged 6–23 months [OR = 4.80; 95% CI [3.57, 6.46]), stunted children (AOR = 1.69; 95% CI [1.20, 2.38]), and children in middle wealth quintile households (AOR = 1.92; 95% CI [1.16, 3.17]) and among children living in the Terai ecological region compared with mountains (AOR = 3.48; 95% CI [2.02, 5.99]). Low‐grade inflammation is associated with obesity or overweight; nonetheless, our analysis did not show any association between ferritin and obesity/overweight.

There was an increased risk of vitamin A deficiency among children living in the central (AOR = 3.41; 95% CI [1.38, 8.41]), mid‐western (AOR = 2.82; 95% CI [1.14, 6.98]) and eastern (AOR = 3.52; 95% CI [1.49, 8.35]) regions compared with the western region. In addition, the odds were higher among children belonging to households that reported severe food insecurity compared with food‐secure households (AOR = 2.02; 95% CI [1.15, 3.54]) and in children who had not consumed other fruits in the previous 24 h (AOR = 2.91; 95% CI [1.05, 3.48]). We did not find association between vitamin A status and consumption of vitamin A‐rich fruits and vegetables. We found that the odds of vitamin A deficiency increased in breastfed children aged 6–23 months (AOR = 1.92; 95% CI [0.58, 6.25]) but there was statistical significance. There was no association between age and vitamin A deficiency in our study.

The odds of zinc deficiency were higher among children living in rural area than urban (AOR = 2.69; 95% CI [1.26, 5.77]) and higher among the lowest wealth quintile compared with the second (AOR = 0.45; 95% CI [0.31, 0.64]), middle (AOR = 0.60; 95% CI [0.40, 0.93]) and Fourth (AOR = 0.49; 95% CI [0.30, 0.76]) quintiles. We did not find association between Zinc and neither HAZ nor Stunting. There was no association between age and Zinc deficiency in our study.

## 4 DISCUSSION

To our knowledge, our study is the most extensive analysis to explore individual, household, and national risk factors of iron, vitamin A and zinc deficiency using a nationally representative sample of children aged 6–59 months in Nepal. The risk factors for the different micronutrients had limited similarities. Individual risk factors included children aged 6–23 months, stunted children were more likely to be iron deficient. Children who had not eaten other fruits were more likely to be vitamin A deficient. On household risk factors, children in the middle‐wealth quintile were more likely to be iron deficient than those in the poorest quintile, while those belonging to severely food insecure households and the poorest wealth quintile were more likely to be vitamin A and Zinc deficient, respectively. The national risk factors indicate that iron deficiency odds were greater if children lived in the Terai than the mountains and belonged to specific ethnic groups (Newar, other Terai caste and Muslims) compared with the Janajati ethnic group.

Children aged 6–23 months were four times more at risk of iron deficiency (based on ferritin) than their male counterparts aged 24–59 months. Our findings are comparable to earlier studies by Chandyo et al. (2015) in Bhaktapur, Siegel et al. (2006) in South Central Nepal, and Villalpando et al. (2003) in Mexico, indicating the likelihood of iron deficiency was very high in children below 24 months of age. Rapid growth in infants, particularly those born premature or low birth weight, have higher iron requirements, possibly explaining the drop in iron stores (Moreno‐Fernandez et al., 2019; Soh et al., 2004). As the infant grows and blood volume expands, an increased amount of iron is needed in haemoglobin (Wharf et al., 1997). Increased lean body mass will also require iron for both myoglobin and enzymes. Iron deficiency is the only disorder of iron balance in which nutrition has the primary role (Lynch et al., 2018). An important but unanswered question is to know the prevalence of ID and associated risk factors among children below 6 months.

Stunted children had almost twice the risk to develop iron deficiency (measured by both ferritin and sTfR) compared with their non‐stunted counterparts. The nationally representative study by Habib et al. (2016) in Pakistan based on 7138 children showed that stunting is a risk factor for iron deficiency anaemia. A study of 569 children in central Terai, Nepal, showed stunting as a risk factor for iron deficiency in the bivariate analysis, but the effect was not in the multivariate analysis (Siegel et al., 2006). Siegel's study was not nationally representative and consisted of a narrower age band compared with our study. Iron deficiency results in decreased appetite (Lawless et al., 1994), leading to reduced intake of other growth limiting factors, such as energy and protein. We expected the association between zinc status and stunting, but instead, we did not observe this in our study. Many questions remain unanswered, such as the causal direction of stunting and ID and the possible mechanism that explains this interaction in under 2 years children.

Our study showed a high risk of ID among middle‐income households compared with the poorest households in general. Our findings contradict in Europe and Alaofè et al., 2017 in northern Benin, which showed lower socioeconomic status increased ID risk. Our study contributes to the body of knowledge given that the study in Benin is much smaller than ours, and the European study occurs in a developed setting. A study has shown that middle‐income households feed unhealthy snack foods and beverages (USFB) thus displacing other nutrient‐rich foods (Maunder et al., 2015). A study among children 12–23‐month‐old in Kathmandu Valley has shown that snack foods and beverages are unhealthy due to poor nutrient profiles. These snack foods contribute 5.2%, 21.5% and 46.9% of total energy intake (TEI) for children in the low, moderate, and high tertiles of USFB consumption, respectively (Pries et al., 2017). A past study found that while DD score generally improve with higher maternal education and household wealth, the consumption of packaged food products also increased for the higher SES groups (Agrawal et al., 2019). The underlying mechanism that increases ID risk among middle‐income households compared with the poorest households warrants further research to understand the role of USFB and other potential risk factors.

An unexpected high ID risk was observed among Brahmin/Chettri, and other castes (Newar, other Terai caste and Muslims) compared with the Janajati caste. Caste is a Hindu construct that determines, among other factors, vocation, economic viability, and social hierarchy. Pandey et al. (2013), showed that the Janajati and Dalit had the most significant proportion of women in the lowest wealth quintile. There is a need for further studies to understand what protects Janajati from ID risk despite their position in the lowest wealth quintile.

Our study shows that children with low socioeconomic status had a greater risk of low serum Zn concentrations than their high socioeconomic status counterparts. Our findings agree with Lartey et al. (2000) in Ghana and Villalpando et al. (2003) in Mexico that showed the risk of low serum zinc levels was more remarkable in children of low socioeconomic level. Children from wealthier households consume foods high in Zinc, have lower intestinal infections and consume food lower in phytates, thus improving absorption (Gibson, 2006). A dose–response was not observed in the association between zinc deficiency and socioeconomic status in our study. Future studies could explore reasons why the wealthiest households are not protected from Zn deficiency compared with the poorest households.

Children residing in rural settings were at higher risk of low serum Zn concentrations than their urban counterparts. Our study findings agree with those seen by Sharma and Yadav (2019) in Allahabad district, Uttar Pradesh and Galetti et al. (2016) in rural Benin. Children living in rural locations have inadequate zinc levels in their diet due to limited access to foods rich in Zinc, such as animal products and oysters. (Gibson, 2006). Rural households rely on plant‐based sources of Zn such as whole grains, nuts and beans. However, zinc assimilation from plant‐based sources depends on the soil zinc content, and its bioavailability is influenced by inhibitors such as phytic acid, calcium, and perhaps polyphenols (Lönnerdal, 2000). We did not see any association between serum Zinc and Stunting. Due to periods of rapid growth, children are at an increased risk of deficiency, leading to stunting (Stammers et al., 2015). A study on the association between the zinc content of plant‐based foods and soil zinc content will be essential to address deficiencies in rural areas.

Nonconsumption of other fruits and vegetables was associated with vitamin A deficiency. Our findings are consistent with previous studies in Ethiopia (Herrador et al., 2014), Benin (Alaofè et al., 2017) and Brazil (Osorio et al., 2004), which revealed that intake of low fruit and vegetable contributes to hidden hunger. Other fruits and vegetables should still be a significant source of provitamin A carotenoids, such as b‐carotene and b‐cryptoxanthin primarily obtained from plant sources.

Households who reported severe food insecurity were associated with increased vitamin A deficiency. Additional analysis also revealed that vitamin A was significantly less prevalent among children aged 6–23 months who consumed five or more food groups (Table S1). Our findings are not consistent with previous results in Brazil, showing that food insecurity was not associated with vitamin A deficiency (Gubert et al., 2016). We speculate that low HFI leads to deprived sources of vitamin A required throughout the life cycle. At infancy, colostrum and human milk are rich sources of preformed vitamin A and provitamin A carotenoids (Haskell & Brown, 1999). After infancy, vitamin A is obtained from animal and plant sources. However, further studies are needed to learn better how to target food‐insecure households, given that Vitamin A deficiency is no longer a public health problem in Nepal.

Our study did not show an association between consumption of animal source foods such as dairy products, flesh foods, and eggs with neither vitamin A, Zinc nor Iron status. Animal‐source foods are the richest source of absorbable Zinc, most notably the organs and flesh of mammals, fowl, fish, and crustaceans (King et al., 2016). Vitamin A is obtained as preformed vitamin A esters in rich sources like liver, milk, cheese, eggs or food products fortified with vitamin A (Tanumihardjo et al., 2016). Iron is richly available in muscle tissue as haem iron in the form of haemoglobin and myoglobin. Haem iron is estimated to contribute up to 40% or more of the total absorbed iron (Institute of Medicine, 2001). Our findings point to an important area of further research to establish the boundaries of where and when the association between animal source foods and micronutrient deficiencies in a population is observed.

The present study reports the prevalence of multiple micronutrient deficiencies among children 6–59 months. The concurrent prevalence of zinc deficiency and ID was the highest (3.3%). The public health significance of this finding is uncertain because there are no guidelines from the World Health Organization (WHO) on the acceptable levels of multiple concurrent deficiencies in populations or their health significance. We recommend further studies to identify at‐risk groups for concurrent deficiencies to guide nutrition interventions and tailor nutrition screening in Nepal, as this is beyond the scope of this study.

The strength of our study is the use of a nationwide population‐based data set that provides a large sample size and statistical power to study the risk factors for hidden hunger among children under 5 in Nepal. However, there are some limitations; first, the cross‐sectional study design limits causal inference. Second recall bias may have influenced our findings because self‐reported data were collected and analysed. Third, it is difficult to establish a clear temporal association between the risk factors and the micronutrient deficiencies due to the study's cross‐sectional design. Lastly, reverse causality is common with cross‐sectional data, such as those of the NNMSS.

## 5 CONCLUSION

Our study highlighted that factors associated with hidden hunger in Nepal are younger and stunted children, diets and inequities by region, caste, household poverty, rural residence and HFI. To address these risk factors, the Nepalese government should focus on improving the diets of young and stunted children through healthy diet counselling and improving national food systems (food production, processing, retail preparation and consumption). Social cash transfers could address national and household risks by providing purchasing power to poorest and food‐insecure households in high‐risk development regions. Further studies could focus on implementation quality and impact of micronutrient control programmes to accelerate progress. There is a need to shed more light on at‐risk groups for concurrent micronutrient deficiencies.

## CONFLICT OF INTERESTS

The authors declare that there are no conflict of interests.

## AUTHOR CONTRIBUTIONS

Stanley Chitekwe, Naveen Paudyal, Karan Courtney Haag, and Andre Renzaho conceptualize and prepared the manuscript. Kedar Raj Parajuli provided overall guidance to the manuscript. Kingsley Agho and Abukari Issaka did the study design and data analysis and prepared manuscript. All authors reviewed and provided input into the manuscript.

## Supporting information

Supporting information.

## Data Availability

The data that support the findings of this study are available from the corresponding author upon reasonable request.
