# Supplementary material for: Individual, household and national factors associated with iron, vitamin A and zinc deficiencies among children aged 6–59 months in Nepal
Source: Matern Child Nutr. 2021 Dec 13;18(Suppl 1):e13305. doi: 10.1111/mcn.13305 (PMC8770653; doi:10.1111/mcn.13305)
Supplement: Supplementary file 1 — Supporting information. [file MCN-18-e13305-s001.pdf]

Supplementary Table 1. Descriptive statistics of the serum concentrations of ferritin, soluble transferrin receptor (sTfR), retinol binding protein (RBP) and zinc in children aged 6-59 months in Nepal

Biomarker†	Mean (SD)	Quartiles			n
		Q25	Q50	Q75	
Ferritin	26.1 (19.6)	11.4	21.6	35.7	1652
sTfR	9.4 (5.5)	6.2	7.4	10.3	1652
RBP	1.0 (0.3)	0.8	1.0	1.2	1652
Zinc	89.2 (37.4)	65.2	83.9	107.4	1560

†Adjusted for inflammation using the BRINDA approach (Namaste, Rohner, et al., 2017).

Supplementary Table 2. Dietary Diversity (≥5 food groups) by the micronutrient status and socio-economic factors.

**Characteristic**	**Dietary Diversity ≥5**		**P-value**	**Unadjusted OR (95% CI)**	**p-value**
	**%**	**95%CI**			
**BRINDA Adjusted Ferritin**					
No	26.1	[22.3,30.2]		1.00	
Yes	33.9	[27.3,41.2]	*	1.45 (1.07, 1.97)	0.017
**BRINDA Adjusted sTfR**					
No	25.7	[21.5,30.4]		1.00	
Yes	32.6	[26.9,38.8]	*	1.40 (1.03,1.92)	0.035
**BRINDA Adjusted Zinc**					
No	28.9	[25.3,32.7]		1.00	
Yes	26.4	[19.4,34.8]		0.88 (0.62, 1.25)	0.473
**Vitamin A**					
No	29.2	[25.3,33.4]		1.00	
Yes	17.7	[10.6,28.0]	*	0.52 (0.28, 0.97)	0.039
**Ferritin & sTfR**					
No	27.0	[23.1,31.2]		1.00	
Yes	33.2	[26.3,41.0]		1.35 (0.98, 1.86)	0.065
**Ferritin & sTfR & Zinc**					
No	28.0	[24.3,32.1]		1.00	
Yes	33.9	[20.5,50.4]		1.31 (0.68, 2.53)	0.408
**Ferritin & sTfR & Zinc & Vit A**					
Yes	#	#		#	#
No	#	#		#	#
**Household Wealth Index**					
Poorest	15.0	[11.1,20.1]		1.00	
Poorer	21.7	[16.4,28.1]		1.56 (0.94, 2.57)	0.078
Middle	26.0	[21.5,31.1]	***	1.99 (1.31, 3.03)	0.002
Richer	33.1	[26.6,40.3]		2.79 (1.78, 4.38)	< 0.001
Richest	46.5	[37.9,55.3]		4.91 (3.01, 8.00)	< 0.001

For Peer Review

**Mother's education**					
No schooling	14.9	[9.9,21.9]		1.00	
Primary	22.1	[14.7,31.9]	***	1.62 (0.87, 2.99)	0.125
Secondary education or more	37.4	[31.6,43.7]		3.41 (1.93, 6.03)	< 0.001

* p<0.05; **p<0.01; ***p<0.001 by chi-squared test. #values and 95%CI too wide due to small sample size.

For Peer Review
